# Predictors of persistent postsurgical pain following total knee arthroplasty: A protocol for systematic review and meta-analysis

**DOI:** 10.1080/24740527.2019.1614881

**Published:** 2019-07-30

**Authors:** Vahid Ashoorion, Behnam Sadeghirad, Li Wang, Anthony Adili, Rachel Couban, Gordon Guyatt, Jason Busse

**Affiliations:** a The Michael G. DeGroote Institute for Pain Research and Care, McMaster University, Ontario, Canada; b Isfahan Medical Education Research Centre, Isfahan University of Medical Sciences, Isfahan, Iran; c Department of Health Research Methods, Evidence, and Impact, McMaster University, Ontario, Canada; d Department of Anesthesia, McMaster University, Ontario, Canada; e The Michael G. DeGroote Centre for Medicinal Cannabis Research, McMaster University, Ontario, Canada; f Department of Surgery, McMaster University, Ontario, Canada

**Keywords:** knee replacement, chronic pain, risk factors, prognosis, meta-analysis

## Abstract

**Background**: Total knee arthroplasty (TKA) is a commonly performed procedure, primarily when knee joints have been damaged by progressive arthritis; however, over 20% of surgical patients develop persistent postsurgical pain (PPSP). We plan to conduct a systematic review and meta-analysis of factors associated with the development of PPSP following TKA.

**Methods**: We will include peer-reviewed cohort or case–control studies that explore, in an adjusted model, factors associated with the development of PPSP after TKA. We will identify eligible studies, in any language, by a systematic search of MEDLINE, EMBASE, CINAHL, AMED, Scopus, SPORTDiscus, and PsycINFO, from inception of each database. Pairs of reviewers will, independently and in duplicate, screen titles and abstracts of identified citations, review the full texts of potentially eligible studies, and extract information from eligible studies. When possible, we will pool estimates of association for all independent variables reported by more than one study and report both an adjusted odds ratio and the absolute risk increase and associated 95% confidence intervals (Cis). We will use the Grading of Recommendations Assessment, Development and Evaluation (GRADE) approach to summarize the quality of evidence for all meta-analyses as high, moderate, low, or very low.

**Discussion**: Our results will facilitate identification of patients at risk for the development of PPSP following TKA, highlight promising predictors for further study, and help guide the design of interventional studies to improve prognosis of high-risk patients.

## RÉSUMÉ

**Contexte**: L’arthroplastie complète du genou est une intervention communément réalisée, principalement lorsque les articulations des genoux ont été endommagées par l’arthrite dégénérative; toutefois, plus de 20 % des patients ayant été opérés développent de la douleur postopératoire persistante. Nous comptons ffectuer une revue systématique et une méta-analyse des facteurs associés au développement de la douleur postopératoire persistante après une arthroplastie complète du genou.

**Méthodes**: Nous incluerons les études de cohorte ou les études cas-témoins examinées par des pairs qui explorent, dans un modèle ajusté, les facteurs associés au développement de la douleur postopératoire persistante après une arthroplastie complète du genou. Nous recenserons les études admissibles, peu importe la langue dans laquelle elle sont écrites, en faisant une recherche systématique dans MEDLINE, EMBASE, CINAHL, AMED, Scopus, SPORTDiscus et PsycINFO, depuis les tout débuts de chaque base de données. Des paires d’examinateurs passeront en revue les titres et les résumés des citations répertoriées de manière indépendante et en double, puis ils examineront les textes complets des études potentiellement admissibles et en extrairont l’information. Lorsque possible, nous ferons une estimation globale de l’associatio pour toutes les variables indépenantes rapportées par plus d’une étude et rapporterons les rapports de cote ajustés, ainsi que l’augmentation du risque absolu et les IC à 95 % associés. Nous utiliserons l’approche GRADE pour résumer la qualité des données probantes pour toutes les méta-analyses, afin de déterminer si elle est élevée, modérée, faible ou très faible.

**Discussion**: Nos résultats faciliteront le repérage des patients à risque de développer de la douleur postopératoire persistante après une arthroplastie complète du genou, mettront en lumière les prédicteurs prometteurs pour de futures et contribuerot à orienter la conception d’études interventionnelles afin d’améliorer le pronostic des patients à haut risque.

## Introduction

Total knee arthroplasty (TKA) is one of the most common orthopedic surgeries performed worldwide, primarily for patients with advanced osteoarthritis who have failed nonoperative treatment.^1,2^ In 2016, more than 67 000 patients underwent TKA in Canada.^3^ The prevalence of osteoarthritis is increasing, due to higher rates of obesity and increasing life expectancy in Western societies,^4^ and from 2005 to 2030 the number of TKAs performed in the United States is expected to grow by more than six times.^5^

Pain is the primary reason for patients to undergo knee replacement with the expectation that surgery will provide relief^6^; however, more than 20% of patients develop persistent postsurgical pain (PPSP), with higher rates associated with revision surgery.^7–10^ Moreover, despite advances in surgical technology and perioperative anesthetic management, the incidence of PPSP after TKA surgery has not decreased.^11^

We found eight reviews that have explored predictors of PPSP following TKA, of which three were narrative^12–14^ and five were systematic reviews^15–19^ (Table 1). The systematic reviews all had important limitations, including outdated searches and failure to evaluate the overall quality of evidence.^14–19^ The two systematic reviews that reported meta-analyses ignored nonsignificant risk factors when the measures of association were not reported, which risks overestimating the magnitude of associations. Statistical pooling in both reviews was problematic. One review used Fisher’s *Z* effect size for pooling estimates of association from a variety of statistical tests (i.e., correlation, *t* test, analysis of variance, chi-square, linear regression, and logistic regression).^14^ Interpretation of results pooled using Fisher’s *Z* is nonintuitive, and pooling estimates from a variety of statistical analyses with different properties is inappropriate. The other review that provided pooled estimates only looked at procedural and surgical techniques as predictors of anterior knee pain following primary TKA, combined both unadjusted and adjusted estimates of association, and pooled treatment effects from randomized controlled trials with measures of association from observational studies.^19^ We propose to conduct a new systematic review and meta-analysis of observational studies to identify predictors of PPSP following TKA that addresses limitations of prior reviews.

**Table 1.** Characteristics of prior systematic reviews on predictors of PPSP after TKA.

Citation	Eligible study designs	Search period	Databases searched	Assessed risk of bias	Assessed overall certainty in evidence	Type of synthesis
Harmelink et al.^15^	Observational studies	January 2000 and January 2016	EMBASE and MEDLINE	Yes	Yes	Narrative
Vissers et al.^16^	Observational studies with at least 6 weeks’ follow-up (included both total knee and total hip arthroplasty)	From database inception to January 2011	MEDLINE and EMBASE	Yes	No	Narrative
Wylde et al.^17^	Cohort studies	From database inception to October 2016	MEDLINE, EMBASE and PsycINFO	Yes	No	Narrative
Lewis et al.^18^	Cohort, case–control, or cross-sectional studies	From 1980 to December 2012	MEDLINE, EBSCO, Scopus, CINAHL, SPORTDiscus, and AMED	Yes	No	Pooled adjusted and nonadjusted measures of association
Duan et al.^19^	RCTs or observational studies	From database inception to July 25, 2017	MEDLINE, EMBASE, and Cochrane Central	Yes	No	Pooled adjusted and crude odds ratios from RCTs and observational studies

PPSP = persistent postsurgical pain; TKA = total knee arthroplasty; RCT = randomized controlled trial.

## Methods

### Standardized reporting

We registered our protocol with PROSPERO (CRD42018065943) and will follow the Meta-analysis of Observational Studies in Epidemiology Statement for reporting of our systematic review.^20^

### Data sources and search strategy

We will systematically search MEDLINE, EMBASE, CINAHL, AMED, Scopus, SPORTDiscus, and PsycINFO, from inception of each database, without any language restriction. An experienced medical librarian (R.C.) has developed search strategies for each database (Appendix). We will review reference lists from eligible studies and related reviews for additional potentially eligible studies.

### Eligibility criteria and study selection

We will include peer-reviewed cohort and case–control studies that enroll adults (18 years or older) who undergo TKA and investigate, in an adjusted analysis, risk factors for PPSP after TKA. Studies will be ineligible if their predictive models include significant associations with variables collected after baseline, because the association may be a result of PPSP. When study populations overlap by more than 50% among eligible articles, we will include only the study with the largest sample size and longest follow-up. We will exclude conference abstracts.

Pairs of reviewers will screen the titles, abstract, and full-text articles of potentially eligible studies, independently and in duplicate. Reviewers will, when necessary, resolve disagreements by discussion or by consultation with an adjudicator. We will use online systematic review software (Distiller SR, Evidence Partners [Internet]. Ottawa (ON), Canada; https://www.evidencepartners.com/products/distillersr-systematic-review-software/) to facilitate literature screening and prepare aPreferred Reporting Items for Systematic Reviews and Meta-Analyses (PRISMA) flow diagram to illustrate the flow of studies through the selection process.

### Data extraction

Pairs of reviewers, working independently and in duplicate, will extract relevant information from all eligible studies. Before starting data abstraction, we will conduct calibration exercises to ensure consistency between reviewers. We will use piloted, standardized forms to extract the following information: (1) study characteristics (e.g., authors, publication year, country of origin, funding source); (2) study population characteristics (e.g., sample size, age, sex distribution, underlying condition leading to TKA, measure of disease severity, opioid use prior to surgery); (3) surgical procedure (e.g., unilateral or bilateral, primary or revision surgery, cemented, type of prosthesis); (4) risk of bias and statistical analysis approaches; and (5) measures of association with PPSP for all independent risk factors explored in an adjusted model.

When a study reports more than one regression model, we will use the model with the largest population, longest follow-up, or largest number of risk factors. When studies report the dependent variable (PPSP) as both a dichotomous outcome (e.g., presence or absence of PPSP) using logistic regression and as a continuous outcome (e.g., pain score) using linear regression, we will use the results from the logistic regression model. All disagreements on data extraction will be resolved through discussion. We will contact authors for clarification of eligibility or missing data.

### Risk of bias assessment

We will use the following criteria from the *Users’ Guides to the Medical Literature* to assess the risk of bias: (1) representativeness of study population; (2) validity of outcome assessment; (3) proportion of missing data and loss to follow-up (≥20% will be considered high risk of bias); and (4) whether predictive models are appropriately adjusted.^21^ We define a model as appropriately adjusted when it includes age, sex, and a measure of disease severity (e.g., pain intensity, type of injury, grade of osteoarthritis). We will assess whether the adjusted model was data driven (only those with significant associations in bivariate analysis were entered in the final model) or theory driven (all risk factors of interest were entered).

### Data synthesis

We will assess interrater agreement of full-text screening with the kappa statistic.^22^ We will report intensity of PPSP across studies as the median and interquartile range, after converting all pain scales to a 10-cm visual analogue scale.^23,24^ For continuous predictors that are entered as categorical variables in the regression model (i.e., multiple odds ratios [ORs] reported for one variable), we will assume linearity and that the associations across categories are independent of each other and calculate the OR and 95% confidence interval (CI) for each category using Bucher’s approach and combine ORs using the inverse variance method to produce a single OR for the predictor.^25,26^ In studies that excluded predictors from their final adjusted analysis due to nonsignificant association in univariate or bivariate analysis or the adjusted OR and its 95% CI were not reported due to nonsignificant association in final adjusted model, we will use an OR of 1 and impute the associated variance using the hot deck approach to avoid overestimation of association.^26,27^

We will pool all independent factors assessed for an association with PPSP that are reported by more than one study as an OR and associated 95% CI and calculate the absolute risk increase. We will estimate the baseline risk for PPSP after TKA using the lowest rate of PPSP from the study eligible for review with the largest sample size among studies at low risk of bias. When the measure of association is reported as relative risk, we will convert it to an OR using the reported baseline risk in the reference or unexposed group (participants without the risk factor).^28^ We will use DerSimonian-Laird random effects models for all meta-analyses.^29^ We will assess publication bias by visual assessment of funnel plots and Egger’s test when at least ten studies are included in a meta-analysis.^30,31^

If pooling is not possible, we will explore the consistency of association between pooled results and studies reporting the same predictors that could not be pooled. We will define nonpoolable predictors as promising if they meet the following criteria: (1) a statistically significant association with PPSP of *P* ≤ 0.01, (2) a large magnitude of association (OR ≥ 2.0), and (3) a sample size of at least 500 patients. Data analysis will be performed using STATA software (Version 15.1).

### Subgroup analyses, metaregression, and sensitivity analyses

Statistical tests of heterogeneity can be misleading when estimates of precision are very narrow due to large sample sizes; thus, we will evaluate heterogeneity for all pooled estimates through visual inspection of forest plots.^32^ We will explore four a priori hypotheses to explain variability between studies, assuming a larger association with PPSP with the following study characteristics: (1) longer duration of follow-up, (2) higher threshold for PPSP (e.g., moderate to severe pain va. no to mild pain), (3) larger proportion of patients lost to follow-up, and (4) greater risk of bias on a criterion-by-criterion basis. In addition, we will conduct a subgroup analysis exploring studies that adjusted for preoperative pain vs. those adjusted with another measure of disease severity (e.g., grade of Osteoarthritis (OA)), and if we find a significant subgroup effect we will prioritize pooled measures of association from studies that adjusted for pain severity. We will conduct subgroup analyses only if each subgroup contains three or more studies and explore for subgroup effects with a test of interaction.^33^ We will examine the effect of imputing data for nonsignificant predictors and converting categorical data for patient age to continuous data in a sensitivity analysis.

### Certainty of evidence

We will use the Grading of Recommendations Assessment, Development and Evaluation (GRADE) approach to assess the certainty of evidence as high, moderate, low, or very low, based on risk of bias, inconsistency, indirectness, imprecision, and publication bias.^34^ Once we have established the baseline risk of PPSP after TKA, we will estimate the absolute increase in risk for both modifiable and nonmodifiable factors that would alter clinical decision making and rate down for imprecision if the 95% CI associated with the risk difference includes this threshold.

## Discussion

Due to the large volume of TKAs performed each year and the high rate of patients who develop PPSP, there is an urgent need for a high-quality systematic review to identify factors associated with the development of persistent pain. Modifiable factors that show large associations have the potential to be directly targeted to reduce the rate of PPSP after TKA. Moreover, patients who present with important nonmodifiable factors may benefit from nonspecific interventions. Either scenario would help direct clinical trials to establish the effectiveness of promising strategies.

Our proposed review has several strengths in relation to prior reviews. First, we will update the search. Second, we will pool similar predictors across studies and optimize interpretability by reporting measures of association in both relative and absolute measures. Third, we will incorporate nonsignificant predictors into our meta-analyses by imputing measures of association when they are not reported to avoid overestimating the strength of associations. Fourth, we will assess the overall certainty of evidence using the GRADE approach.

A potential limitation is the quality and comprehensiveness of reporting in the primary literature. We may find that nonmodifiable factors, such as disease severity or surgical approaches, have been commonly explored for their association with PPSP after TKA, whereas modifiable factors, such as anxiety or coping abilities, have not. The findings of our review will help inform patients considering TKA about their prognosis and identify key areas for future research.

## Supplementary Material

Supplemental Material
