# Supplementary material for: Predictors of persistent postsurgical pain following total knee arthroplasty: A protocol for systematic review and meta-analysis
Source: Can J Pain. 2019 Jul 30;3(2):10–5. doi: 10.1080/24740527.2019.1614881 (PMC8730639; doi:10.1080/24740527.2019.1614881)
Supplement: Supplemental Material [file UCJP_A_1614881_SM8262.pdf]

Appendix: Search strategies for Medline and EMBASE

Medline

1. exp Chronic Pain/
2. chronic pain.mp.
3. (ongoing adj3 pain).mp. [mp=title, abstract, original title, name of substance word, subject heading word, floating sub-heading word, keyword heading word, protocol supplementary concept word, rare disease supplementary concept word, unique identifier, synonyms]
4. (persist* adj3 pain).mp. [mp=title, abstract, original title, name of substance word, subject heading word, floating sub-heading word, keyword heading word, protocol supplementary concept word, rare disease supplementary concept word, unique identifier, synonyms]
5. (long term adj3 pain).mp. [mp=title, abstract, original title, name of substance word, subject heading word, floating sub-heading word, keyword heading word, protocol supplementary concept word, rare disease supplementary concept word, unique identifier, synonyms]
6. (long lasting adj3 pain).mp. [mp=title, abstract, original title, name of substance word, subject heading word, floating sub-heading word, keyword heading word, protocol supplementary concept word, rare disease supplementary concept word, unique identifier, synonyms]
7. or/1-6
8. exp Pain, Postoperative/
9. (post surgery adj3 pain).mp. [mp=title, abstract, original title, name of substance word, subject heading word, floating sub-heading word, keyword heading word, protocol supplementary concept word, rare disease supplementary concept word, unique identifier, synonyms]
10. (postoperat* adj3 pain).mp. [mp=title, abstract, original title, name of substance word, subject heading word, floating sub-heading word, keyword heading word, protocol supplementary concept word, rare disease supplementary concept word, unique identifier, synonyms]

11. or/8-10

12. 7 or 11

13. exp Arthroplasty, Replacement, Knee/

14. total knee arthroplasty.mp.

15. total knee replacement.mp.

16. total knee prosthesis.mp.

17. TKA.mp.

18. TKJR.mp.

19. or/13-18

20. 12 and 19

21. exp Epidemiologic Studies/

22. exp case control studies/

23. exp cohort studies/

24. exp Cross-Sectional Studies/

25. case control.ti,ab.

26. (cohort adj (study or studies or analys*)).ti,ab.

27. ((follow up or observational or uncontrolled or non randomi#ed or nonrandomi#ed or epidemiologic*) adj (study or studies)).ti,ab.

28. ((longitudinal or retrospective or prospective or cross sectional) and (study or studies or review or analys* or cohort*)).ti,ab.

29. 21 or 22 or 23 or 24 or 25 or 26 or 27 or 28

30. 20 and 29

31. 30

32. limit 31 to humans

EMBASE

1. exp chronic pain/
2. chronic pain.mp.
3. (ongoing adj3 pain).mp. [mp=title, abstract, heading word, drug trade name, original title, device manufacturer, drug manufacturer, device trade name, keyword, floating subheading word, candidate term word]
4. (persist* adj3 pain).mp. [mp=title, abstract, heading word, drug trade name, original title, device manufacturer, drug manufacturer, device trade name, keyword, floating subheading word, candidate term word]
5. (long term adj3 pain).mp. [mp=title, abstract, heading word, drug trade name, original title, device manufacturer, drug manufacturer, device trade name, keyword, floating subheading word, candidate term word]
6. (long lasting adj3 pain).mp. [mp=title, abstract, heading word, drug trade name, original title, device manufacturer, drug manufacturer, device trade name, keyword, floating subheading word, candidate term word]
7. 1 or 2 or 3 or 4 or 5 or 6
8. knee.mp.
9. 7 and 8
10. exp postoperative pain/

11. (post-surgery adj3 pain).mp. [mp=title, abstract, heading word, drug trade name, original title, device manufacturer, drug manufacturer, device trade name, keyword, floating subheading word, candidate term word]
12. (postoperat* adj3 pain).mp. [mp=title, abstract, heading word, drug trade name, original title, device manufacturer, drug manufacturer, device trade name, keyword, floating subheading word, candidate term word]
13. 10 or 11 or 12
14. exp total knee arthroplasty/
15. exp total knee arthroplasty/
16. total knee arthroplasty.mp.
17. total knee replacement.mp.
18. total knee prosthesis.mp.
19. TKA.mp.
20. TKJR.mp.
21. 14 or 15 or 16 or 17 or 18 or 19 or 20
22. exp clinical study/
23. exp case control study/
24. family study/
25. longitudinal study/
26. prospective study/
27. cross-sectional study/
28. cohort analysis/
29. follow-up/

30. cohort*.ti,ab.

31. (cohort adj (study or studies or analys*)).ti,ab.

32. case control.ti,ab.

33. ((follow up or observational or uncontrolled or non randomi#ed or nonrandomi#ed or epidemiologic*) adj (study or studies)).ti,ab.

34. ((longitudinal or retrospective or prospective or cross sectional) and (study or studies or review or analys* or cohort*)).ti,ab.

35. 22 or 23 or 24 or 25 or 26 or 27 or 28 or 29 or 30 or 31 or 32 or 33 or 34

36. animals/ not humans/

37. nonhuman/

38. exp Animal Experiment/

39. exp Experimental Animal/

40. animal model/

41. exp Rodent/

42. (rat or rats or mouse or mice).ti.

43. 36 or 37 or 38 or 39 or 40 or 41 or 42

44. 9 or 13

45. 21 and 44

46. 35 and 45

47. 46 not 43
